# Breastfeeding status and determinants of current breastfeeding of Syrian refugee children in Turkey

**DOI:** 10.1186/s13006-022-00538-w

**Published:** 2023-02-01

**Authors:** Siddika Songül Yalcin, Esin Aydin Aksoy, Suzan Yalcin, Mehmet Ali Eryurt

**Affiliations:** 1 Department of Pediatrics, Faculty of Medicine, Hacettepe University, Ankara, Turkey; 2 Departmant of Social Pediatrics, Institute of Child Health, Hacettepe University, Ankara, Turkey; 3 Department of Food Hygiene and Technology, Faculty of Veterinary Medicine, Selçuk University, Konya, Turkey; 4 Institute of Population Studies, Hacettepe University, Ankara, Turkey

**Keywords:** Breastfeeding, Refugee women, Preceding birth interval, Prelacteal feeding

## Abstract

**Background:**

Turkey hosts the highest number of refugees in the World including 65% of Syrian refugees who reside in Turkey. Mothers and children were the most negatively affected among the Syrian refugees who had to migrate from their countries as a result of the civil war in Syria. One of the most important issues in terms of child health is breastfeeding. Breastfeeding in migrants should be promoted worldwide to mitigate infant mortality and diseases. The aim of this study is to examine the association between breastfeeding status in Syrian refugee children under two years and socio-demographic characteristics of Syrian refugee mothers with further analysis of Turkey Demographic and Health Survey-Syrian Migrant-2018 (TDHS-SM-2018) data.

**Methods:**

The data source is the TDHS-SM-2018. Data for the last-born children with a gestational duration greater than 32 weeks from the mothers’ singleton pregnancy, aged less than two years old and living with the mother were included (unweighted *n* = 744). The dependent variable was the breastfeeding status (breastfeeding in the last 24 h during the study period) in children under two years. Complex sample logistic regression evaluated the associations.

**Results:**

The percentage of breastfeeding in children under the age of two years was found to be 62.4%, and the total median breastfeeding duration was 14.6 months. Univariate analysis showed that the earlier mothers immigrated to Turkey, the higher the current breastfeeding rate. Breastfeeding rates were found to be higher among people living in the South and East regions (65.2% and 65.1% respectively). Multivariable binary complex sample logistic regression revealed that breastfeeding status at the study period was associated with long preceding birth interval; delivery in a public hospital; absence of prelacteal feeding; being non-pregnant; and the region and age of the child. No relationship for current breastfeeding was found with maternal activities, maternal life satisfaction, financial satisfaction, and educational status.

**Conclusion:**

Current breastfeeding in our sample was more likely among mothers with a longer birth interval who avoided prelacteal feeding. The Baby-Friendly approach and family planning services should be integrated into refugee health centers.

## Background

All the persons are not affected equally by emergency situations; infants are more vulnerable to natural and social disasters than others [1]. The feeding strategy for infants and young children should be to create an environment that encourages intensive breastfeeding of children up to the age of at least two years, to solve any problems and to ensure the continuity of breastfeeding in emergencies, including migration [2]. However, the humanitarian crisis caused by internal conflict for years and forced migration in Syria have affected infant health negatively [3, 4].

Migration to many countries has been experienced after the civil war in Syria and 65% of the refugees came to Turkey [5]. There were 3,674,588 Syrian refugees under temporary protection stats in Turkey. Fifteen point three per cent of Syrians living in Turkey are aged 0–4 years [5]. Approximately 200,000 Syrian babies were born in Turkey between 2011 and 2016 [6]. The infant mortality rate is 22 per thousand and the under-five mortality rate is 27 per thousand according to the Turkish Demographic Health Survey-Syrian Migrant-2018 (TDHS-SM-2018) data [7]. On the other hand, mortality rates in Syrian children were found to be lower in Turkey compared to the rates for the preceding ​​five years and ten years which were spent in Syria [7]. This might be due to the healthcare services and free vaccination opportunities in Turkey [5]. Breastfeeding is also important for children to have better health outcomes including reduced mortality, but breastfeeding rates in Syria are quite below the global averages [8]. Due to the high fertility rates (5.3 children born to Syrian migrant women in Turkey) [7], more studies are needed to increase breastfeeding follow-up and supports to reduce infant-child mortality in the Syrian migrant population. Inequalities in breastfeeding rates of migrant and non-migrant women, depending on their birthplaces, length of stay in the host country, and acculturation have been reported in the literature [9, 10]. For this reason, it is important to examine breastfeeding rates according to migration status in various cultures, to identify inter-communal differences, and to promote policies to improve breastfeeding.

There are limited studies that evaluate the factors affecting breastfeeding in Syrian migrant children [7, 11]. According to TDHS-SM-2018, the median breastfeeding duration is 13.7 months and 40% of children under the age of two are breastfed appropriately for their age in Turkey [7]. However, the socio-economic and bio-demographic characteristics of the breastfed mother-infant pairs in refugees were not analyzed. The aim of this study is to examine the status of breastfeeding of Syrian children under two years old, together with associated factors including socio-demographic characteristics of Syrian refugee women and mother-infant dyad characteristics, with further analysis of TDHS-SM-2018 data for Turkey, which hosts the highest number of Syrian refugees in the world. This study is the first comprehensive study to assess the relationship between socio-demographics; cultural characteristics; maternal activities; life visions; and maternal prenatal and infant perinatal characteristics; and current breastfeeding status among Syrian refugee children in Turkey as far as we know. The results of this study can be used in future refugee crises and the policies to improve breastfeeding.

## Methods

### Data source

The data source of the study is TDHS-SM-2018, which was conducted by the Hacettepe University Institute of Population Studies.

#### Survey objectives

The TDHS-SM-2018 is designed to produce estimates of important demographic characteristics and health indicators for Syrians living in Turkey. The survey aims to produce the first ever household level mother and child health indicators of the Syrian refugee population in Turkey.

#### Sample design

A weighted, multi-stage, stratified cluster sampling approach was used in the survey. The framework on the Syrian population in Turkey was obtained from the Ministry of Interior, Presidency of Migration Management. Because of the unavailability of a frame that included each Syrian household in Turkey, a sample was created based on the population size of each quarter, which is Turkey’s smallest administrative unit. Stratification in the survey considered only one variable for the camp / non-camp population. The first stage of sample selection comprised selecting quarters from each stratum as primary sampling units. Following the creation of block lists by field teams that identified Syrian homes in the chosen quarters, the second stage of sample selection — the selection of households — was conducted. In order to interview 2000 households (target sample size) in 100 clusters for the TDHS-SM-2018 sample, 20 households from each cluster were chosen. All women between the ages of 15 and 49 who usually resided in the chosen households and / or were present there the night before the interview were considered to be eligible for the women’s questionnaire.

#### Fieldwork

The fieldwork was conducted between 23 November 2018 to 12 February 2019. Data collection for the TDHS-SM-2018 sample was carried out in teams. Each team consisted of six people who are bilingual in Turkish and Arabic: three female interviewers, a male measurer, a female listing personnel and a team supervisor. Before the interviews were conducted, the listing personnel scanned the households in the determined cluster and listed the households where Syrian refugees live. Then, one of the female interviewees interviewed women from the households which were selected from the listed households. Since measuring instruments are heavy, men are preferred as the measurer and the male measurer obtained the height and weight of women aged 15–49.

#### Data processing

In the survey, the technique of computer-assisted personal interviewing in which a computer or a tablet is used were applied. CSPro software (U.S. Census Bureau) was employed for the data processing.

In the TDHS-SM-2018, 2216 women aged 15–49 living in 1826 households were successfully interviewed on issues related to child health for all children under five years, together with socio-economic and bio-demographic characteristics of the Syrian refugees living in Turkey. Hacettepe University Ethics Commission reviewed and approved the TDHS-SM-2018 and informed consent was obtained from all interviewed women in the original study.

#### Inclusion criteria

In this further analysis, the last children with a gestational duration greater than 32 weeks from the mothers’ singleton pregnancy, born in the two years before the research date and living with the mother were enrolled (unweighted n = 744).

#### Study parameters

The dependent variable of our study was “breastfeeding in the last 24 hours during the study period”.

As independent variables, the sociodemographic characteristics of family members [the place of residence in Turkey (non-camp and camp), region (central, east, west and south), highest education level of mother and father (no education, primary, secondary, higher), mothers’ literacy in Arabic (cannot read at all, able to read only parts of the sentences, able to read the whole sentence), mothers’ reading and writing abilities in Turkish, household size (< 6, 6–9 and ≥ 10 members), the presence of other wives of husband, smoking status of the household members], the economic status [existence of a person in the household working in a paid job, and existence of a person in the household receiving any payment of benefits], the maternal characteristics [maternal age at the first birth, and the current pregnancy status at the interview, mother’s body mass index (BMI) values, life satisfaction, satisfaction with financial status and social activities], the antenatal, birth and postnatal characteristics of enrolled children [mother’s desire for child when pregnant (soon: wanted at the time of conception, later: after became pregnant; does not want), mothers’ age at birth of the current child, the birth order, the preceding birth interval, the number of antenatal care attendances, the gestational duration of the child, the country where they gave birth (Turkey and Syria), the place of delivery (home, public hospital and private hospital), the type of delivery (Cesarean and vaginal delivery), child’s birth weight, sex, and age, starting time of breastfeeding (early initiation of breastfeeding (EIBF) and after), prelacteal feeding (giving the baby any fluid or nutrients, including sugared water, honey, tea, milk other than breast milk, infant formula, plain water, except breast milk in the first three days after birth), the presence of bottle feeding, and the presence of postnatal care in the two months after the birth were taken from TNSA-SM data.

In addition, the duration since migration to Turkey coded as “23 months before the birth of the child or after the birth”, “2– 3 years before the birth of the child”, “4–5 years before the birth of the child” and “at least 6 years before the birth of the child”.

In the study, a ten-point scale of satisfaction with the financial situation of the household was used to evaluate subjective economic well-being. Similarly, mother’s satisfaction with life is measured again using a ten-point scale: 1–10. One point denoted the worst case, 10 the best case. This variable was recoded into two categories ≥ 5 for satisfied, < 5 for dissatisfied. The social activities of the mothers were evaluated according to their exercise habits, visiting friends at home, using internet and watching television programs for women. Exercise habits information was collected with the question, “Do you do any physical activity such as sports, walking?“.

#### Statistical analyses

In Demographic Health Surveys (DHS), as in most surveys, the sample is selected with unequal probability to increase the number of cases available (and hence decrease sample variability) for specific locations or subgroups for which data are required. Weights are used when statistics are tabulated in order to provide the appropriate representation, and corrections for varying response rates are made when weights are calculated because of sample design [12]. Unweighted and weighted numbers of cases and percentage distributions of the data were calculated. Breastfeeding percentages according to case characteristics were calculated using a weighted database.

Using complex sample logistic regression, the difference between breastfeeding percentages according to household characteristics, maternal activities and antenatal and postnatal characteristics of last-born child were examined, and univariate odds ratios (OR) and 95% confidence interval (CI) were calculated.

The difference between the percentages of breastfeeding in the weighted sample for variables including household characteristics, maternal activities and antenatal and postnatal characteristics of the last-born child was analyzed with the Chi-square test. Variables with a difference of *p* < 0.10 were determined and included for multivariable binary logistic regression analysis. Among these variables, it was observed that there was a relationship between private hospital deliveries, frequency of antenatal follow-up, and the financial status of the household, and “delivery place” was further analyzed. There was a relationship between prelacteal feeding and bottle usage, and prelacteal feeding was selected for further analysis. Therefore, multivariable binary complex sample logistic regression analysis evaluated region (South vs. West, Central vs. West, East vs. West), respondent currently working (yes vs. no), currently pregnant (yes vs. no or unsure), place of delivery (home vs. public sector, private sector vs. public sector), history of prelacteal feeding (yes vs. no), preceding birth interval (first child vs. ≥ 36 months, < 18 months vs. ≥ 36 months, 18–35 vs. ≥ 36 months), duration between migration and birth of the child (2–3 vs. < 2 years., 4–5 vs. < 2, ≥ 6 vs. < 2 years) and child’s age in months as independent variables on breastfeeding.

Value of median breastfeeding duration was determined by linear interpolation of percentage of first group (beginning with the birth group) below 0.5 and previous group percentage using the following formula: median = 𝑚𝑖−1 + [(𝑝𝑖−1 − 0.5) / (𝑝𝑖 −1−𝑝𝑖)] ∗ (𝑤𝑖) where 𝑝𝑖 is the proportion breastfeeding for the first group where the proportion is below 0.5, *pi*-1 is the proportion breastfeeding for the preceding group, mi-1 is the midpoint value for the preceding group, and wi (two months) is the time width of the group taken as the difference between the midpoint value of the current group and the preceding group [12].

Analyses were performed with Stata 13 and SPSS 22 package program.

## Results

### Family characteristics

In weighted data, 730 Syrian mothers who had immigrated to Turkey and had children under the age of two were included in the study. About three-quarters of mother-infant dyads lived in the South and East regions of Turkey and 3.8% lived in refugee camps; and 21.6% shared the same house with 10 or more people. Of the mothers, 30% gave birth to their first child when they were under the age of 18 years. Maternal age at birth was less than 20 years old in 27% of the last-born children. While 10.9% of the women had no education, 18.4% were illiterate in their own language. Only 11.4% of women had reading and writing abilities in Turkish. Almost none of the women (95.5%) had a job, while 69.7% of the husbands had a job and 72.9% of the households had at least one working person. More than half of the households (54.5%) were receiving donations. The husbands of 5.8% of the women had another wife. While 5.9% of women were smokers, more than half of the households (61.9%) had at least one smoker at home (Table 1). One quarter of the women (24.8%) were satisfied with their household income, while two out of three (65.3%) were satisfied with their lives (Tables 1 and 2).

**Table 1:** Characteristics of household and association with current breastfeeding status of the last-born children under two years of age: percentages and OR results

	**Number and percentage of children**		**Currently breastfed children**			
	Unweighted N (%)^a^	Weighted N (%)^b^	N (%)^b^	*P* ^c^	OR^d^	95% CI
**Total**	744	730	456 (62.4)			
**Region of Turkey**				0.068		
West	130 (17.5)	138 (18.9)	73 (52.9)		1.00	
South	276 (37.1)	270 (37.0)	176 (65.2)		1.67	1.23, 2.25
Central	58 (7.8)	61 (8.4)	37 (60.7)		1.33	0.80, 2.21
East	280 (37.6)	261 (35.7)	170 (65.1)		1.66	1.19, 2.31
**Place of residence**				1.000		
Non-camp	665 (89.4)	702 (96.2)	439 (62.5)		1.00	
Camp	79 (10.6)	28 (3.8)	18 (64.3)		1.03	0.68, 1.57
**Household size, person**				0.621		
< 6	253 (34.0)	247 (33.8)	149 (60.3)		1.00	
6–9	332 (44.6)	325 (44.6)	209 (64.3)		1.19	0.87, 1.62
≥ 10	159 (21.4)	158 (21.6)	98 (62.4)		1.09	0.74, 1.62
**Husband has other wives**				0.289		
No	698 (93.8)	688 (94.2)	426 (62.0)		1.00	
Yes	46 (6.2)	42 (5.8)	30 (71.4)		1.55	0.75, 3.22
**Mother’s age at the first birth, year**				0.382		
< 18	217 (29.2)	218 (29.9)	143 (65.6)		1.26	0.88, 1.78
18–19	203 (27.3)	198 (27.1)	126 (63.6)		1.17	0.82, 1.67
≥ 20	324 (43.5)	314 (43.0)	188 (59.9)		1.00	
**Mother’s age at birth of the child, year**				0.761		
< 20	197 (26.5)	200 (27.5)	122 (61.0)		0.89	0.64, 1.23
20–29	408 (54.8)	400 (54.8)	255 (63.8)		1.00	
≥ 30	139 (18.7)	130 (17.8)	79 (61.2)		0.90	0.59, 1.36
**Mother’s highest education level**				0.203		
No education	82 (11.0)	80 (10.9)	42 (52.5)		1.00	
Primary	287 (38.6)	283 (38.7)	181 (64.2)		1.64	0.93, 2.88
Secondary	316 (42.5)	310 (42.5)	194 (62.6)		1.53	0.91, 2.58
Higher	59 (7.9)	57 (7.9)	39 (68.4)		2.01	0.96, 4.23
**Mother’s literacy in Arabic**				0.160		
Cannot read at all	138 (18.5)	135 (18.4)	78 (58.2)		1.00	
Able to read only parts of sentence	94 (12.6)	91 (12.5)	65 (70.7)		1.74	0.99, 3.06
Able to read whole sentence	512 (68.8)	504 (69.0)	314 (62.3)		1.19	0.80, 1.78
**Mother can read and write in Turkish**				0.330		
No	659 (88.6)	647 (88.6)	401 (62.0)		1.00	
Yes	85 (11.4)	83 (11.4)	56 (67.5)		1.22	0.75, 2.00
**Father’s highest education level**						
No education	79 (10.6)	77 (10.5)	45 (58.4)	0.741	1.00	
Primary	295 (39.7)	293 (40.1)	188 (64.2)		1.30	0.80, 2.10
Secondary	287 (38.6)	279 (38.2)	178 (63.8)		1.27	0.76, 2.15
Higher	75 (10.1)	75 (10.3)	43 (57.3)		0.98	0.51, 1.89
Not known	8 (1.1)	6 (0.8)	4 (66.7)		1.14	0.22, 6.01
**Mother’s current working status**				0.092		
No	709 (95.3)	697 (95.5)	441 (63.3)		1.00	
Yes	35 (4.7)	33 (4.5)	15 (46.9)		0.52	0.25, 1.11
**Husband’s current working status, last week**				0.746		
Yes	242 (32.5)	221 (30.3)	140 (63.3)		0.95	0.67, 1.35
No	502 (67.5)	509 (69.7)	316 (62.1)		1.00	
**Household had at least one working person**						
No	214 (28.8)	198 (27.1)	129 (65.2)	0.361	1.00	
Yes	530 (71.2)	532 (72.9)	327 (61.5)		0.85	0.62, 1.18
**Household receiving donations**						
No	329 (44.2)	332 (45.5)	207 (62.3)	0.897	1.00	
Yes	415 (55.8)	398 (54.5)	250 (62.8)		1.02	0.78, 1.32
**Financial satisfaction with the household income**				0.075		
Dissatisfied: < 5	557 (74.9)	549 (75.2)	353 (64.3)		1.00	
Satisfied: ≥ 5	187 (25.1)	181 (24.8)	103 (56.9)		0.74	0.52, 1.04
**Smoking mother**				0.499		
No	700 (94.1)	687 (94.1)	428(62.3)		1.00	
Yes	44 (5.9)	43 (5.9)	29(67.4)		1.24	0.70, 2.21
**Anyone smokes at home**				0.248		
No	290 (39.0)	278 (38.1)	181 (65.1)		1.00	
Yes	454 (61.0)	452 (61.9)	275 (60.8)		0.83	0.59, 1.17

*Abbreviations: CI* Confidence interval, *OR* Odds ratio

^a^Column percentage

^b^weighted row percentage

^c^Chi-square test

^d^Univariate binary complex sample logistic regression analysis

One out of eight mothers (12.1%) was exercising. Two thirds of women (61.3%) were using the internet. The BMI value for 23.7% of women was 30 kg / m^2^ or more (Table 2). Of women who had children under the age of two, 14.2% were currently pregnant (Table 3).

**Table 2:** Maternal activities and association with current breastfeeding status of the lastborn children under two years of age: percentages and OR results

	**Number and percentage of children**		**Currently breastfed children**			
	Unweighted N (%)^a^	Weighted N (%)^b^	N (%)^b^	*P* ^d^	OR^e^	95% CI
**Do any exercise**						
No	658 (88.4)	642 (87.9)	399 (62.1)	0.494	1.00	
Yes	86 (11.6)	88 (12.1)	58 (65.9)		1.16	0.65, 2.07
**Organise home meetings**				0.421		
No	377 (50.7)	379 (51.9)	242 (63.9)		1.00	
Yes	367 (49.3)	351 (48.1)	214 (61.0)		0.89	0.64, 1.25
**Use internet**				0.903		
No	295 (39.7)	283 (38.8)	176 (62.2)		1.00	
Yes	449 (60.3)	447 (61.2)	280 (62.6)		1.01	0.75, 1.38
**Watch women’s programs on television**				0.256		
No	532 (71.5)	523 (71.6)	320 (61.2)		1.00	
Yes	212 (28.5)	207 (28.4)	136 (65.7)		1.21	0.88, 1.67
**Mother’s life satisfaction**				0.197		
Dissatisfied: < 5	259 (34.8)	253 (34.7)	150 (59.3)		1.00	
Satisfied: ≥ 5	485 (65.2)	477 (65.3)	306 (64.2)		1.23	0.86, 1.77
**Mother’s BMI** ^**c**^ **, kg / m** ^**2**^				0.296		
< 25	330 (44.4)	319 (43.7)	209 (65.5)		1.00	
25–29	228 (30.6)	227 (31.2)	134 (59.0)		0.75	0.52, 1.10
≥ 30	174 (23.4)	173 (23.7)	107 (61.8)		0.86	0.59, 1.22

*Abbreviations*: *CI* Confidence interval, *BMI* Body mass index, *OR* Odds ratio

^a^Column percentage

^b^weighted row percentage

^c^missing data: 12

^d^Chi-square test

^e^univariate binary complex sample logistic regression analysis

**Table 3:** Antenatal and postnatal characteristics of last-born child and association with current breastfeeding status of the last-born children under two years of age: percentages and OR results

	**Number and percentage of children**		**Currently breastfed children**			
	Unweighted N (%)^a^	Weighted N(%)^b^	N (%)^b^	*P* ^d^	OR^e^	95% CI
**Migration date to Turkey according to birth of the child**				< 0.001		
Within 23 months before the birth or after birth	128 (17.2)	134 (18.4)	70 (52.2)		1.00	
2–3 years before birth	226 (30.4)	229 (31.4)	130 (56.8)		1.20	0.74, 1.94
4–5 years before birth	314 (42.2)	304 (41.6)	206 (67.8)		1.93	1.26, 2.95
6–10 years before birth	76 (10.2)	63 (8.6)	51 (81.0)		3.93	2.02, 7.63
**Birth country of child**				< 0.001		
Turkey	708 (95.2)	692 (94.8)	444 (64.2)		3.59	1.52, 8.49
Syria	36 (4.8)	38 (5.2)	13 (34.2)		1.00	
**Birth order of the child**				0.264		
1	189 (25.4)	191 (26.2)	112 (58.6)		1.00	
2–3	345 (46.4)	343 (47.0)	214 (62.4)		1.18	0.76, 1.82
≥ 4	210 (28.2)	196 (26.8)	130 (66.7)		1.42	0.86, 2.34
**Mother’s desire for child when pregnant**				0.847		
Soon	626 (84.1)	614 (84.1)	384 (62.5)		1.00	
Later	42 (5.7)	42 (5.8)	25 (59.5)		0.87	0.44, 1.70
Does not want	76 (10.2)	74 (10.1)	48 (64.9)		1.15	0.67, 1.97
**Preceding birth interval, months**				0.028		
First birth	189 (25.4)	191 (26.2)	112 (58.6)		0.69	0.43, 1.12
< 18	123 (16.5)	122 (16.7)	65 (53.3)		0.56	0.35, 0.89
18–35	259 (34.8)	250 (34.2)	167 (66.8)		1.00	0.67, 1.50
≥ 36	173 (23.3)	167 (22.9)	112 (67.1)		1.00	
**Antenatal care attendance, number**				0.146		
< 4	250 (33.6)	251 (34.4)	163 (64.9)		1.00	
4–7	276 (37.1)	265 (36.3)	171 (64.5)		0.97	0.68, 1.39
≥ 8	218 (29.3)	214 (29.3)	122 (57.0)		0.71	0.51, 1.00
**Gestational duration**				0.756		
8 months	42 (5.6)	39 (5.3)	23 (59.0)		0.86	0.45, 1.63
9 months	702 (94.4)	691 (94.7)	434 (62.8)		1.00	
**Place of delivery**				0.001		
Home	21 (2.8)	22 (3.0)	10 (43.5)		0.39	0.16, 0.97
Public sector	618 (83.1)	598 (82.0)	394 (65.9)		1.00	
Private sector	102 (13.7)	106 (14.5)	52 (49.1)		0.50	0.33, 0.76
Missing	3 (0.4)	4 (0.5)				
**Cesarean delivery**				0.208		
No	552 (74.2)	543 (74.4)	332 (61.1)		1.00	
Yes	192 (25.8)	187 (25.6)	124 (66.3)		1.24	0.90, 1.71
**Birth weight** ^**d**^ **, g**				0.363		
< 2500	104 (14.0)	102 (14.0)	59 (57.8)		0.72	0.46, 1.13
2500–3999	481 (64.7)	475 (65.1)	310 (65.3)		1.00	
≥ 4000	56 (7.5)	54 (7.3)	34 (63.0)		0.91	0.49, 1.69
**Child age, months**				< 0.001		
< 6	229 (30.8)	226 (30.4)	205 (90.7)		1.00	
6–11	193 (25.9)	187 (25.1)	140 (74.9)		0.29	0.15, 0.56
12–17	172 (23.1)	168 (22.6)	81 (48.2)		0.09	0.05, 0.16
18–23	150 (20.2)	149 (20.0)	31 (20.8)		0.03	0.02, 0.05
**Sex of child**				0.366		
Male	411 (55.2)	402 (55.1)	257 (63.9)		1.00	
Female	333 (44.8)	328 (44.9)	199 (60.7)		0.87	0.65, 1.17
**Initiation time of breastfeeding, hours**				0.138		
< 1 (early initiation)	547 (73.5)	544 (74.5)	349 (64.2)		1.00	
≥ 1	197 (26.5)	186 (25.5)	108 (23.6)		0.77	0.54, 1.08
**Prelacteal feeding**				0.009		
No	539 (72.4)	533 (73.1)	349 (65.4)		1.00	
Yes	205 (27.6)	196 (26.9)	108 (54.8)		0.65	0.46, 0.91
**Bottle feeding**				< 0.001		
No	457 (61.4)	451 (61.8)	348 (77.2)		1.00	
Yes	287 (38.6)	279 (38.2)	108 (38.7)		0.19	0.13, 0.26
**Postnatal infant care, within 2 months**				0.391		
No	115 (15.5)	118 (16.1)	78 (66.1)		1.00	
Yes	629 (84.5)	612 (83.9)	379 (61.9)		0.84	0.55, 1.28
**Currently pregnant**				< 0.001		
No or unsure	636 (85.5)	627 (85.9)	443 (70.7)		16.3	8.6, 31.1
Yes	108 (14.5)	103 (14.1)	13 (12.6)		1.0	
**New pregnancy, trimester**				< 0.001		
0	636 (85.5)	626 (85.9)	443 (70.7)			
1	43 (5.8)	40 (5.5)	11 (27.5)			
2	27 (3.6)	26 (3.5)	2 (7.7)			
3	38 (5.1)	37 (5.1)	0 (0.0)			
**Currently breastfeeding**						
No	278 (37.4)	273 (37.5)				
Yes	466 (62.6)	456 (62.5)				

*Abbreviations*: *CI* Confidence interval, *EIBF* Early initiation of breastfeeding, *OR* Odds ratio

^a^Column percentage

^b^weighted row percentage

^c^missing data: 103

^d^Chi-square test

^e^univariate binary complex sample logistic regression analysis

### Child characteristics

Of the children, 94.8% were born in Turkey and 26.8% had the fourth or higher birth order. The preceding birth interval was shorter than 18 months for 16.7% of women. Only 65.5% of women received four or more antenatal care attendances. The majority of mothers (96.6%) gave birth in hospital and the Cesarean section rate was 25.6%. Fourteen per cent of the babies had a birth weight < 2500 g and 55.1% of the children were male. Most babies, (83.9%) were able to obtain postnatal care within two months after birth (Table 3).

### Breastfeeding status according to mother-child characteristics

The frequency of breastfeeding was slightly higher among those living in the southern and eastern regions compared to those living in the western region (*p* = 0.065). There was no significant relationship between mother’s education, literacy status, employment status of the mother, father’s education, employment status of the father, donation received, place of residence (camp vs. city), the size of the household, mother’s satisfaction with financial situation, smoking at home and breastfeeding status of the baby (Table 1). There was no statistical relationship between mother’s social activities, mother’s life satisfaction, mother’s BMI and breastfeeding (Table 2).

It was observed that the ratio of breastfeeding of Syrian children increased significantly as the time spent by mothers in Turkey increased. It was determined that women who had migrated to Turkey at least six years before the birth of the child breastfed approximately four times more often compared to those who gave birth prior to or within 24 months of coming to Turkey (*p* < 0.001; Table 3). The breastfeeding ratio was found to be 3.6 times higher in immigrants who gave birth in Turkey compared to those who gave birth in Syria (*p* < 0.001). Those with a preceding birth interval of less than 18 months were breastfeeding 44% less than those women who had a preceding birth interval of longer than 36 months (*p* < 0.025). Those who gave birth in a private hospital breastfed 50% less when compared with those who gave birth in a public hospital (*p* = 0.001). There was no effect of gestational age, birth order, type of delivery, sex or birth weight of the child on breastfeeding.

Three-quarters (74.5%) of the babies were breastfed within one hour after birth, but one-fourth (26.9%) of them were given prelacteal food, and 38.2% of them fed with a bottle. There was no association between EIBF (*p* = 0.138) and breastfeeding during the study period. Those who were not fed prelacteally (*p* = 0.009) breastfed 1.5 times more than those who were fed prelacteal foods, and those who were not fed with a bottle breastfed 5.2 times more than those who were fed with a bottle (*p* < 0.001, Table 3).

There was no relationship between the baby receiving health care in the first two months after birth and the breastfeeding during the study period for infants under 24 months of age. Breastfeeding was found to occur considerably more in women with no new pregnancy during the study period (*p* < 0.001, Table 3). Breastfeeding ratios seem to be related to the age of the child (*p* < 0.001).

### Breastfeeding in infants under 24 months of age: further analysis

Complex sample binary logistic regression analysis revealed that odds of breastfeeding in South, Central, and East regions are considerably higher compared to the West region: including lower odds of breastfeeding in mothers who are currently pregnant compared to no or unsure; private sector for delivery place compared to public sector; prelacteal feeding compared to no prelacteal feeding; preceding birth interval < 18 months compared to ≥ 36 months; and increasing child age (Table 4).

**Table 4:** Associated factors for current breastfeeding status, multivariable binary complex sample logistic regression analysis

	**Adjusted Odds Ratio**	**95% Confidence Interval**
**Region of Turkey**		
South vs. West	2.02	1.27, 3.21
Central vs. West	2.28	1.00, 5.20
East vs. West	2.01	1.30, 3.11
**Respondent currently working**		
Yes vs. No	0.64	0.27, 1.53
**Currently pregnant**		
Yes vs. no or unsure	0.10	0.05, 0.18
**Place of delivery**		
Home vs. public sector	0.89	0.33, 2.43
Private sector vs. public sector	0.46	0.24, 0.86
**Prelacteal feeding**		
Yes vs. no	0.47	0.30, 0.74
**Preceding birth interval**		
First child vs. ≥ 36 months	0.96	0.52, 1.77
< 18 mo vs. ≥ 36 months	0.51	0.27, 0.96
18–35 mo vs. ≥ 36 months	1.09	0.66, 1.80
**Migration date to Turkey according to the child’s delivery**		
2–3 years before birth vs. within 24 months	0.94	0.55, 1.61
4–5 years before birth vs. within 24 months	0.74	0.44, 1.24
6–10 years before birth vs. within 24 months	0.79	0.36, 1.74
**Child’s age in months**		
One-month increase in age	0.82	0.80, 0.85

### Breastfeeding duration

Total median breastfeeding duration was 14.6 months for the study group. While the median breastfeeding duration is 9 months in cases with bottle feeding, it can be up to 17.7 months in cases without bottle feeding. The median breastfeeding duration was 12.8 months if a baby had a prelacteal feeding and 15.4 months if a baby had not consumed prelacteal food. On the other hand, the median breastfeeding duration was 17.6 months for a non-pregnant woman.

## Discussion

The percentage of breastfeeding in children under the age of 24 months was found to be 62.4 with total median breastfeeding duration 14.6 months for the selected study group including singleton pregnancy and gestational week ≥ 32 weeks. After the war crisis, Syrian refugees settled in the surrounding countries, mostly in Turkey, Lebanon and Jordan [5]. In a study evaluating the nutritional status of Syrian refugees in Jordan, Lebanon and Iraq, it was seen that the breastfeeding ratio in infants aged 0–23 months was 44.5% in the refugee camps in Iraq, while it ranged from 57 to 68% in the refugee camps in Jordan and Lebanon [13]. This result showed that the rate of breastfeeding might vary according to the country. The ratio of breastfeeding of Syrian children increased significantly as the number of years living in Turkey increased. This increase can be attributed to the success of the health service provided to migrants and refugees in Turkey [5, 7]. In our study, 82% of Syrian refugee mothers gave birth in a public hospital and breastfeeding prevalence in those women (65.9%) was more than that of women who gave birth in a private hospital (49.1%). In Lebanon, one of the other countries where Syrian refugees live, most of the births (43.8%) occurred in a private hospital, while the ratio of Syrian refugees who gave birth in a public hospital in Jordan was 51.8%. In Turkey, 99% of deliveries happen in baby-friendly hospitals and this might influence EIBF and then breastfeeding [14, 15]. The rate of exclusive breastfeeding (EBF) in Liberian immigrant women who lived in Ghana for more than eight years was higher than for those who lived in Ghana for fewer than eight years [16]. In a contrasting study from the USA, the breastfeeding ratio of Hispanic immigrants living in the USA for fewer than 5 years was higher than the breastfeeding ratio of immigrants living in the USA for more than 5 years [17]. In a cohort study conducted with 4207 women from the USA, it was shown that every single year lived in the USA decreased the six-month breastfeeding ratio of immigrant women by 4% [18]. In a cohort study of 2704 mother-infant dyads in Hong-Kong, Hong-Kong-born women were found to have a 30% higher risk of stopping breastfeeding early than did immigrants who lived in Hong-Kong for fewer than five years [10].

Acculturation did not have an effect on starting breastfeeding in immigrant women in Germany, but it has been shown that as the length of time they live in the country they migrated to grows, the time they plan to breastfeed decreases and the risk of stopping breastfeeding in the first six months increases. As a different situation, it has been observed that three times more Turkish immigrant women plan to breastfeed for longer than 4 months compared to German women, as the length of time they live in Germany increases [19]. This shows that the cultural characteristics of both the country of residence and the original society are effective in the support for breastfeeding [11, 20]. In further analysis of our study data, it was seen that Syrian refugees’ infants living in the South, Center and East of Turkey were breastfeed two times more often than those living in the West. Syrian refugee mothers living in the border regions of Turkey might have received social support for breastfeeding from people of the same culture.

In the present study infants under 24 months of age who had been born in a public hospital, had higher rates for being breastfed during the study period than those in private facilities. The fact that those who gave birth in a private hospital had a better welfare index, more access to food intake may have played a role here [14, 15]. Similarly, the odds of timely breastfeeding initiation were lower among women who gave birth in private health facilities compared to those who gave birth in public health facilities 0.52 (95% CI 0.36, 0.75) for urban settings in Cambodia [21].

In our study, mothers with a short preceding birth interval (< 18 months) were found to breastfeed less than other mothers. In a study conducted with Saudi mothers, when compared to those who were pregnant for the first time and those with birth intervals less than one year, one year, two years and more than two years; and a positive effect on EBF was found only in those who had a one-year preceding birth interval [22]. On the other hand, approximately one out of seven Syrian women became pregnant again while they had a child under the age of two in our study. Only 13% of pregnant women had continued to breastfeed. The median breastfeeding duration was longer (17.6 months) for a non-pregnant woman. Mothers who breastfed their child for less than 24 months in Ethiopia had shorter birth intervals than mothers who breastfed for more than 24 months [23].

Although the effect of breastfeeding on health is known, it is taboo for some women to continue breastfeeding when they become pregnant [24]. In a study conducted with DHS data from 17 different countries in Africa, Asia and America included, 16.2% of mothers continued to breastfeed their previous children while they were pregnant [25]. In a review that included 19 studies, it was stated that 18% of mothers breastfed until late in their pregnancy, 33% until 28 weeks of pregnancy, and 12.5% ​​throughout their entire pregnancy [26]. Through family planning, pregnancies should be extended to an appropriate period, and babies should be breastfed for at least two years [11, 24]. The World Health Organization (WHO) recommends that the interval between two consecutive pregnancies should be at least two years and the interval between two births at least 33 months in terms of the health of the mother and the child [27]. In TDHS-SM-2018, 43% of Syrian women did not use any method for family planning and 21% of currently married Syrian women had an unmet need for family planning and these women did not use any family planning method although they wanted to interrupt or terminate their deliveries [7]. According to the results of the present study, if the preceding and succeeding birth intervals increased to the range recommended by the WHO, the length of breastfeeding periods for all related children could be extended in Syrian refugee women [11]. Therefore, Syrian refugee women should be informed about family planning with more effective policies in Turkey [11, 20, 24].

In the present study, one-fourth of the Syrian refugee women gave birth by Cesarean section but there was no significant relationship with continued breastfeeding in the first two years. A review of 19 studies including countries located in the Middle East stated that birth by Cesarean section has a rather negative effect on EIBF and giving only breast milk for the first six months [8]. The difference might be related to the limited number of cases and relatively lower Cesarean section rates [28]. In our study, the ratio of EIBF was 74.5%. In the study of the TDHS-SM-2018 data, the ratio of EIBF was 73% and the ratio of EBF in the first six months was 52% [7] and it was in the “good (between 50–89%)” category of the WHO [29]. The ratio of EIBF in Middle Eastern countries was 45% [8]. In a cross-sectional study conducted in Syria in 2014, the ratio of EBF for the first six months for Syrian women was 12.9% [30]. According to these results, the ratio of EIBF and EBF in the first six months is higher in Syrian refugees in Turkey. This can be explained by the fact that most of the hospitals in Turkey are baby friendly hospitals and the ratios of deliveries in the hospital are very high [15]. In the present study, one-fourth of the Syrian migrant mothers fed their babies prelacteally. It was observed that babies who were not fed prelacteally were breastfed 1.5 times more often than babies who were fed prelacteally (OR: 0.65; 95% CI 0.46, 0.91). The median breastfeeding duration was 12.8 months if a baby had a prelacteal feeding and 15.4 months if a baby did have not a prelacteal feeding. Prelacteal feeding percentages were even higher in low-income countries such as Egypt, Ethiopia and Kenya (57.8%, 38.8%, and 26.8%) respectively [31]. In Muslim countries, prelacteal feeding, especially with sugar water, was extensive due to the belief that colostrum has low nutritional value [11, 32–34]. In a study conducted with 4942 mother-infant couples with 2003–2018 DHS data in Turkey, prelacteal feeding was found to be in the range of 29.3–41.4% [35]. Studies have shown a strong relationship between delayed breastfeeding and prelacteal feeding [35–38]. By encouraging EIBF, prelacteal feeding can be prevented and babies can take their first healthy steps. The median breastfeeding duration should be increased by applying the ten baby-friendly hospital rules more widely.

In our study, we observed that Syrian refugee women quickly stopped breastfeeding after six months. It has been observed that babies in the first six months were breastfed 3.4 times more than 6–11 months old babies; 11 times more than 12–17 months old babies; and 33 times more than 18–23 months old babies. According to the TDHS-SM-2018 data, continuity of breastfeeding for one year was 51%, while it decreased to 15% for two years [11, 15]. In another study conducted in Turkey in 2020, 150 Syrian migrant women and baby dyads with an average four years’ stay in Turkey were included and it was shown that the two-year breastfeeding rate was 42% [39]. In addition, in a study with Jordan Family Health and Population Survey data, it was reported that, being younger than 12 months positively affected breastfeeding compared to last-born children aged 12–23 months [24]. Since the education level of refugee women in our country is low, one out of ten Syrian women had never gone to school, and therefore, they may not have fully benefited from breastfeeding training to continue breastfeeding up to two years despite the first breastfeeding initiation in the hospital. As a priority, arrangements should be made to increase the education level of Syrian refugee mothers.

One third of Syrian refugee women fed their babies with a bottle of milk according to data from the present study. Mothers who hadn’t fed their babies with a bottle of milk breastfed 5.2 times more than those who used a bottle for feeding (OR: 0.19; 95% CI 0.13, 0.26). In addition, while the median breastfeeding duration was nine months when bottle feeding, it could be up to 17.7 months in non-bottle feeding. In a study conducted with 2553 mother-infant couples from Canada, the median breastfeeding duration was ten months, and the authors found that even giving breast milk with a bottle reduced the duration of breastfeeding partially [40].

In our study, no statistically significant relationship was found between the social activities that shape the daily life of mothers and the continuation of breastfeeding. It had been observed that breastfeeding was shaped by different dynamics. In our study, 65.3% of mothers stated that they were satisfied with life. We had no queries for breastfeeding satisfaction in the survey questionnaire. Studies analyzing the relationship between maternal satisfaction and breastfeeding are not common yet, but a study from Brazil reported that breastfeeding in the first month after the birth increased maternal satisfaction [41]. Breastfeeding satisfaction is a satisfying feeling that arises from the relationship between mother and baby. In a cross-sectional study of 204 breastfeeding mothers, breastfeeding self-efficacy was reported to be an important factor for breastfeeding satisfaction. High satisfaction was found in 53.4% of breastfeeding mothers [42].

Women who received more antenatal care and gave birth in a private hospital were found to be more satisfied with their financial situation in our study. Socioeconomic status has been shown to be associated with subjective health, well-being, and overall life satisfaction. In a study concluded with 5226 Nepali women in 2018 study, it was reported that women in households with lower wealth status had worse personal health, quality of life, and happiness [43]. However, financial satisfaction did not influence breastfeeding practices, in our study.

### Strengths and limitations

There were some limitations in the present study. A limited number of 730 (weighted) mother-infant couples were included in the study however, it includes a sample of Syrian migrants to Turkey in the years 2017–2018. Follow-up studies with larger samples are needed. Any information about other immigrant groups from other countries and also any comments on unregistered Syrian immigrants living in Turkey couldn’t be given according to the sampling methods used for the present study. Since it is a cross-sectional epidemiological study, cause-effect relationships between dependent and independent factors should be interpreted carefully. The answers we used in our study were based on the mother’s statement only. The mother may have given a biased response on some issues. This is a limitation of all survey studies. There was a mismatch between mothers’ literacy status and graduation from schools. Ninety per cent of women reported that they had at least primary school education, but the literacy rate was 70% in their language. This may be explained by the inability to gain or lose literacy despite going to school, or it may be due to inconsistency in statements. Further studies are needed in this regard. Although interviewers communicated with the mothers in Arabic, due to many dialect differences in Syrian languages, respondents might have experienced some difficulties in understanding and answering interviewers’ questions [5]. This study is the first comprehensive study to evaluate the association between socio-demographic, cultural characteristics, maternal activities, life visions, maternal prenatal, infant perinatal characteristics, and current breastfeeding status among Syrian refugees in Turkey as far as we know.

## Conclusion

The current breastfeeding of Syrian refugees’ children was negatively associated with age of the child, the short preceding birth interval, being pregnant again, delivery in a private hospital, prelacteal feeding and the place of residence in the present study. Breastfeeding status was found to be higher in those living in the south and east regions of Turkey; who gave birth in a public hospital and who did not become pregnant when they had a baby under the age of two. Socio-demographic, cultural characteristics and perinatal factors have a role in the breastfeeding status of Syrian refugees’ children. Integration of baby-friendly approach and family planning services is required in refugee health centers.

## Data Availability

The data that support the findings of this study are available and can be requested from the Hacettepe University Institute of Population Studies (https://hips.hacettepe.edu.tr/en/menu/demographic_and_health_survey_serie-101).

## Abbreviations

BMI: Body mass index
CI: Confidence interval
DHS: Demographic Health Survey
EBF: Exclusive breastfeeding
EIBF: Early initiation of breastfeeding
OR: Odds ratio
TDHS-SM-2018: Turkey Demographic Health Survey-Syrian Migrant Sample-2018
WHO: World Health Organization.
